# Potent Anti-Ovarian Cancer with Inhibitor Activities on Both Topoisomerase II and ^V600E^BRAF of Synthesized Substituted Estrone Candidates

**DOI:** 10.3390/molecules24112054

**Published:** 2019-05-29

**Authors:** Mohamed El-Naggar, Abd El-Galil E. Amr, Ahmed A. Fayed, Elsayed A. Elsayed, Mohamed A. Al-Omar, Mohamed M. Abdalla

**Affiliations:** 1 Chemistry Department, Faculty of Sciences, University of Sharjah, Sharjah 27272, UAE; m5elnaggar@yahoo.com; 2 Drug Exploration & Development Chair (DEDC), Pharmaceutical Chemistry Department, College of Pharmacy, King Saud University, Riyadh 11451, Saudi Arabia; malomar1@ksu.edu.sa; 3 Applied Organic Chemistry Department, National Research Center, Cairo 12622, Egypt; dr_ahmedfayed14@yahoo.com; 4 Respiratory Therapy Department, College of Medical Rehabilitation Sciences, Taibah University, Madinah Munawara 22624, Saudi Arabia; 5 Zoology Department, Bioproducts Research Chair, College of Science, King Saud University, Riyadh 11451, Saudi Arabia; eaelsayed@ksu.edu.sa; 6 Chemistry of Natural and Microbial Products Department, National Research Centre, Cairo 12622, Egypt; 7 Atos Pharma, Elkatyba Land, Belbis 44621, El Sharkya, Egypt; mmostafa201120@yahoo.com

**Keywords:** estrone derivatives, hydrazine, N-substituted pyrazoline, anti-ovarian cancer, topoisomerase II inhibitor, kinase inhibitor

## Abstract

A series of 16-(α-alkoxyalkane)-17-hydrazino-estra-1(10),2,4-trien[17,16-c]-3-ol (**3a**–**l**) and estra-1(10),2,4-trien-[17,16-c]pyrazoline-3-ol derivatives (**4a**–**d**) were synthesized from corresponding arylidines **2a,b** which was prepared from estrone **1** as starting material. Condensation of 1 with aldehydes gave the corresponding arylidine derivatives **2a,b** which were treated with hydrazine derivatives in alcohols to give the corresponding derivatives **3a**–**l**, respectively. Additionally, treatment of **2a,b** with methyl- or phenylhydrazine in ethanolic potassium hydroxide afforded the corresponding N-substituted pyrazoline derivatives **4a**–**d**, respectively. All these derivatives showed potent anti-ovarian cancer both in vitro and in vivo. The mechanism of anti-ovarian cancer was suggested to process via topoisomerase II and ^V600E^BRAF inhibition.

## 1. Introduction

Synthetic alterations of estrone lead to discovering of compounds with diverse biological activities, for example with antitumor effect [1], as anti-breast cancer agent [2] and with antioxidant activity [3]. Estrone derivatives with antitumor activities must be devoid completely of the estrogenic activities [4,5,6]. The inversion of the configuration at C-13 lead to estrone derivatives with antitumor activities devoid from hormonal actions due to conformational change for the overall molecule resulting from the cis junction of rings C and D [4,7]. Some recent publications report on the syntheses and in vitro biological evaluation of several 13α-estrone derivatives [8,9,10,11]. These derivatives exhibited biological activities with substantial anti-proliferative or enzyme inhibitory potentials. Most literature data are mainly focusing on 13α-estrone substitution in ring D, while modified derivatives with ring A substitutions are rarely reported [12]. 

Cancer is considered one of the most significant causes of death worldwide. Ovarian cancer is one of the major causes for death among adult females [13]. Cancer is characterized by abnormal cell proliferation bypassing normal cell growth and death mechanisms. Recently, DNA topoisomerase II inhibitors have been researched as a potential target, which interferes with cancer growth and development [14]. Topoisomerase II inhibition leads finally to cell cycle inhibition and apoptosis in abnormal cancer cells [15]. BRAF kinase inhibition is another possible control mechanism, which was also developed to interfere with cancer cell growth [16]. BRAF kinase constitutes a major signaling process integrated in the RAS-MEK proliferation process. The activation of MEK by BRAF signals finally induces cell proliferation and survival [17]. Inhibiting BRAF activity results in inhibition of the proliferation signals, and thus inhibiting cancer cell development and growth [18].

In view of these observations and in continuation of our previous work in heterocyclic chemistry [19,20,21,22], the current work aimed at evaluating the effect or remote long cage distortion caused by introducing some moieties in both 16 and 17 locations of ring D. We screened some of synthesized estrone candidates for their anti-ovarian cancer potential both in vitro and in vivo. Furthermore, the inhibitory effects of the synthesized compounds were investigated against topoisomerase II and ^V600E^BRAF kinase inhibitors.

## 2. Results and Discussion

### 2.1. Results

#### 2.1.1. Chenical Synthesis

A series of 17-hydrazino-estratrienol (**3a–l**) and pyrazolinestratrienol (**4a–d**) were synthesized from corresponding arylidines **2a,b** which was prepared from estrone **1** as starting material. 

Treatment of aryledine derivatives **2a,b** with hydrazine hydrate, in refluxing methanol or ethanol afforded the corresponding 17-hydrazino-16-α-methoxymethane estrone derivatives **3a,b** and 17-hydrazino-16-α-ethoxyethane estrone derivatives **3c,d**, respectively. 

Also, reaction of compounds **2a,b** with N-methyl hydrazine, in refluxing methanol or ethanol afford 17-N-methyl hydrazino-16-α-methoxymethane estrone derivatives **3e,f** and 17-*N*-methyl hydrazino-16-α-ethoxyethane estrone derivatives **3g,h** respectively. 

**Scheme 1:**
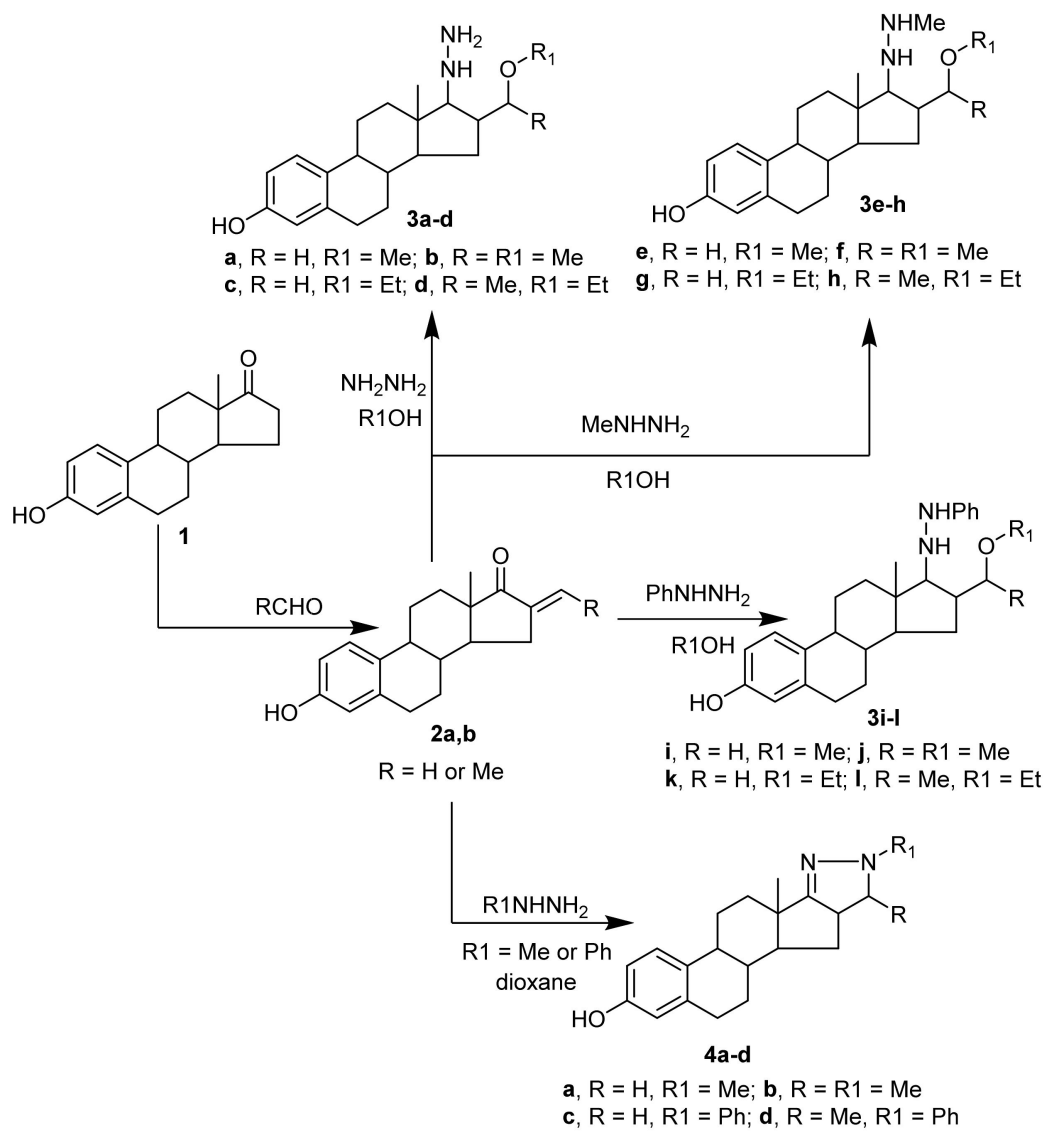
Synthetic route for compounds **2**–**4**.

Additionally, when compounds **2a,b** reacted with *N*-phenyl hydrazine, under the same conditions afford the corresponding 17-*N*-phenylhydrazino- 16-α-methoxymethane estrone derivatives **3i,j** and 17-*N*-phenylhydrazino- 16-α-ethoxyethane estrone derivatives **3k,l**, respectively. Finally, condensation of **2a,b** with CH_3_NHNH_2_ or PhNHNH_2_ in refluxing dioxane afforded the corresponding N-substituted pyrazoline derivatives **4a–d**, respectively (Scheme 1).

#### 2.1.2. Biological Screening

##### In Vitro Cytotoxic Activities

**Figure 1:**
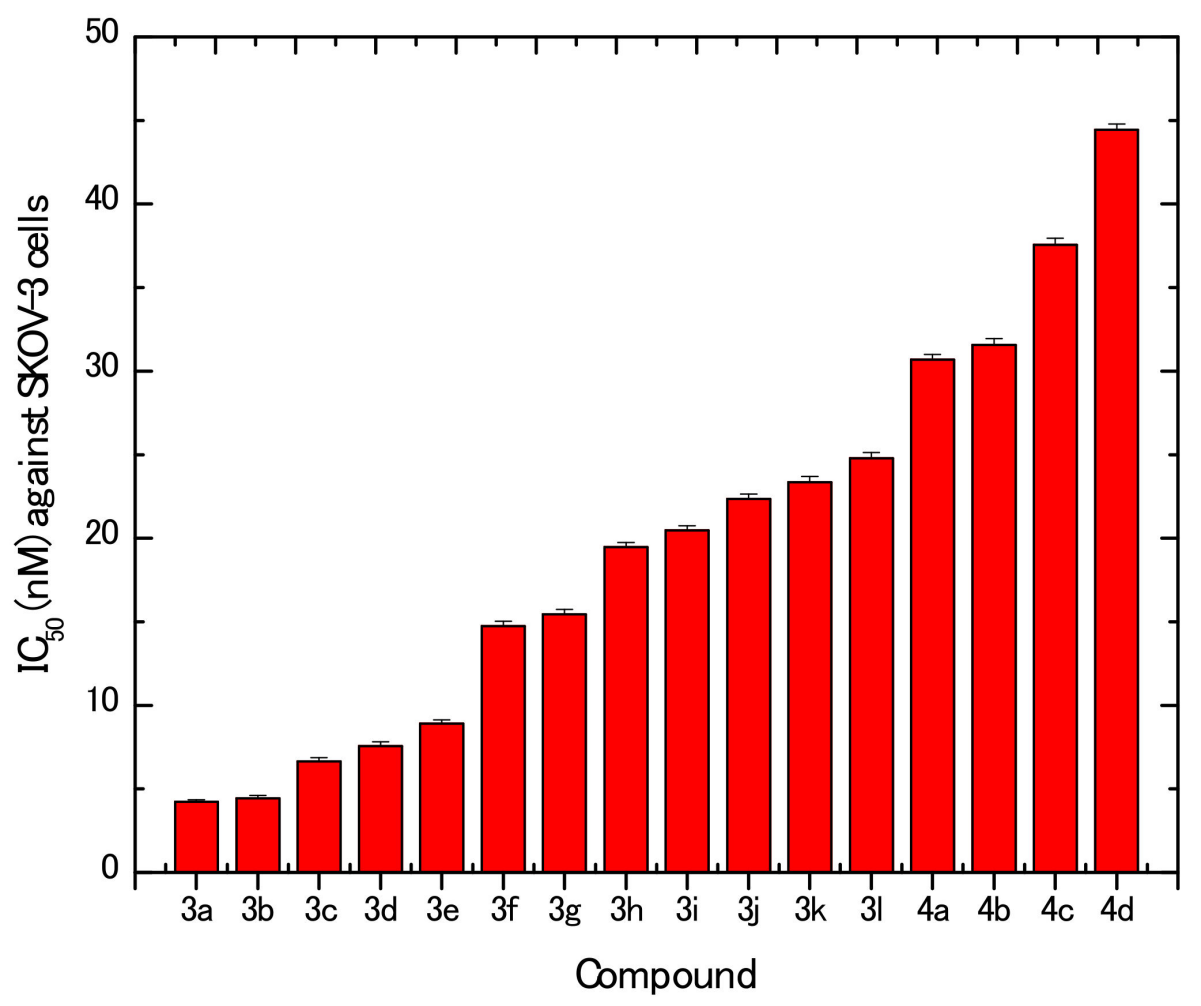
In vitro Cytotoxicity activities of the tested compounds against SKOV-3 cells.

The cytotoxicity of some synthesized derivatives against SKOV-3 cancer cell line in vitro was performed with the MTT assay. The obtained results showed that all synthesized compounds showed potent in vitro cytotoxic activities against SKOV-3 cells at the nanomolar level. Moreover, the effect of the synthesized compounds was dose-dependent. From the obtained IC_50_ values (Figure 1), prepared compounds can be arranged discerningly in the following order: **3a**, **3b**, **3c**, **3d**, **3e**, **3f**, **3g**, **3h**, **3i**, **3j**, **3k**, **3l**, **4a**, **4b**, **4c**, **4d** (Figure 1). Compounds **3a–l** are characterized with the presence of hydrazine group rather than the pyrazoline group present in compounds **4a**–**d**. Furthermore, the alkyl group is found to be more active than the aromatic group. Generally Cpd. **3a** showed highest cytotoxic activity with IC_50_ value of 4.23 ± 0.12 nM, while Cpd. **4d** was the least active, where its IC_50_ value was about 9.5% of that obtained for Cpd. **3a**.

The effect of resveratrol (RES) and doxorubicin (DOX) on SKOV-3 showed IC_50_ of 55 and 250 nM, respectively.

The cytotoxicity of the newly synthesized compounds against fibroblast were as follow **IC_50_ 3a** (1.2 µM), **3b** (2.45 µM), **3c** (5.67 µM), **3d** (7.44 µM), **3e** (8.11 µM), **3f** (9.20 µM), **3g** (11.18 µM), **3h**(16.22 µM), **3i** (15.13 µM), **3j** (16.20 µM), **3k** (17.78 µM), **3l** (20.21 µM), **4a** (23.34 µM), **4b** (24.55 µM), **4c** (25.55 µM), **4d** (30.34 µM). 

##### In Vivo Anti-Ovarian Cancer

**Figure 2:**
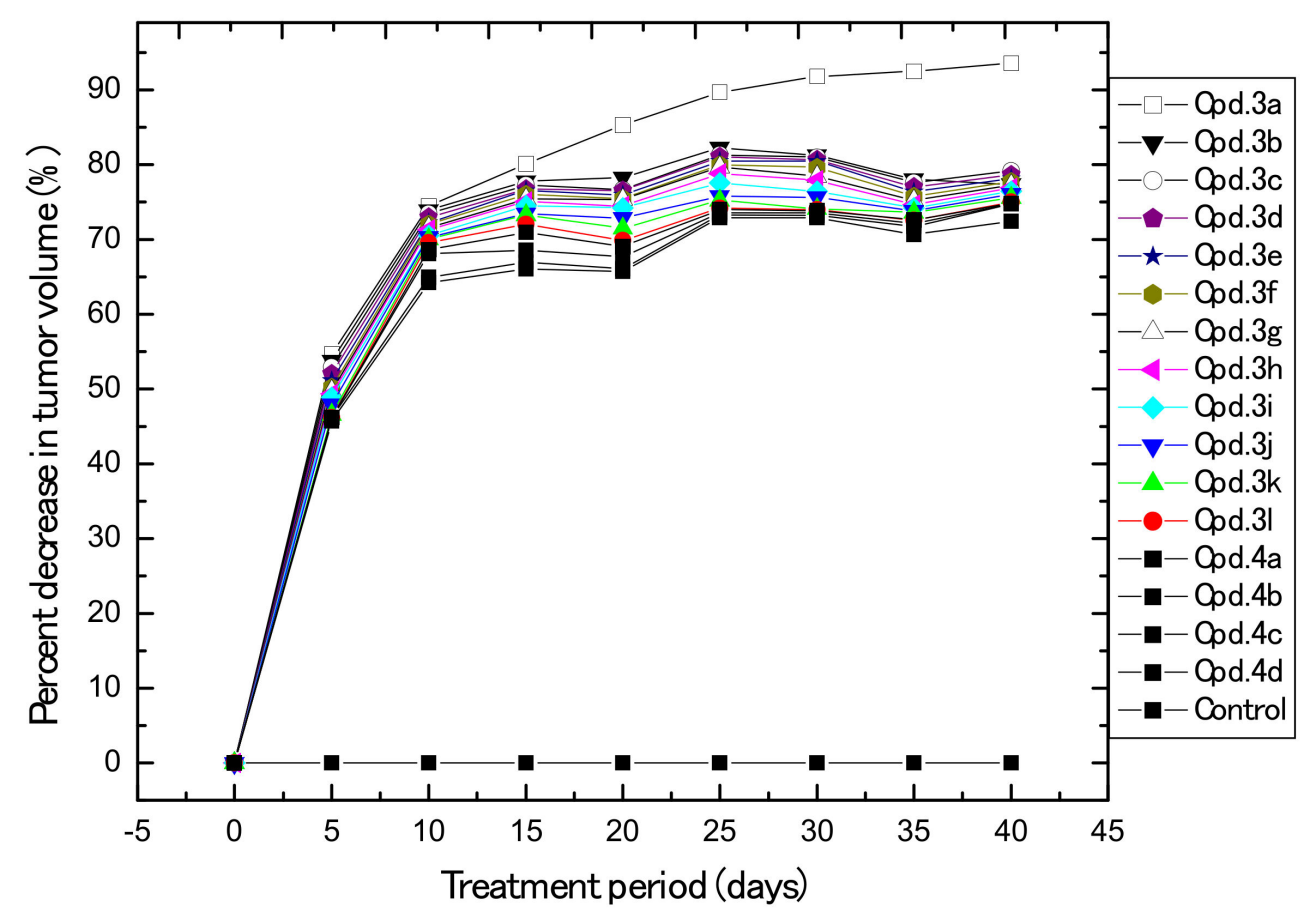
Percentages of decrease in tumor volume in nude mice in response to different synthesized derivatives.

The in vivo anti-ovarian cancer activities of the tested compounds were evaluated using mouse xenograft model. Figure 2 represents the percentages of decrease in tumor growth upon treatment with different synthesized compounds. The obtained results showed that all compounds potentially reduced tumor growth over a period of 40 days of treatment, in comparison to control treatments, which showed normal tumor growth development. Moreover, it can be seen that the reduction in tumor size started directly from the 5th day of treatment, where the reduction percentage ranged from 45.7 ± 0.05% to 54.71 ± 0.01%. The reduction in tumor growth increased significantly reaching its maximal by the end of treatment period (40 days). The highest reduction in tumor growth was recorded for occurred Cpd. **3a**, which significantly decreased tumor growth by about 93.61 ± 0.7%. It can also be seen that Cpd. **3a**, was the most potent for in vivo treatment, where its effect was more obvious starting from day15 of treatment, where the reduction percentage in tumor size due to other compounds was more or less within the same range. We also can see that the in vivo results are coincided with those obtained for *in vitro* results, where the reduction in tumor size followed the same descending order: **3a**, **3b**, **3c**, **3d**, **3e**, **3f**, **3g**, **3h**, **3i**, **3j**, **3k**, **3l**, **4a**, **4b**, **4c**, and **4d**.

#### 2.1.3. Inhibition of Topoisomerase II Activities

**Figure 3:**
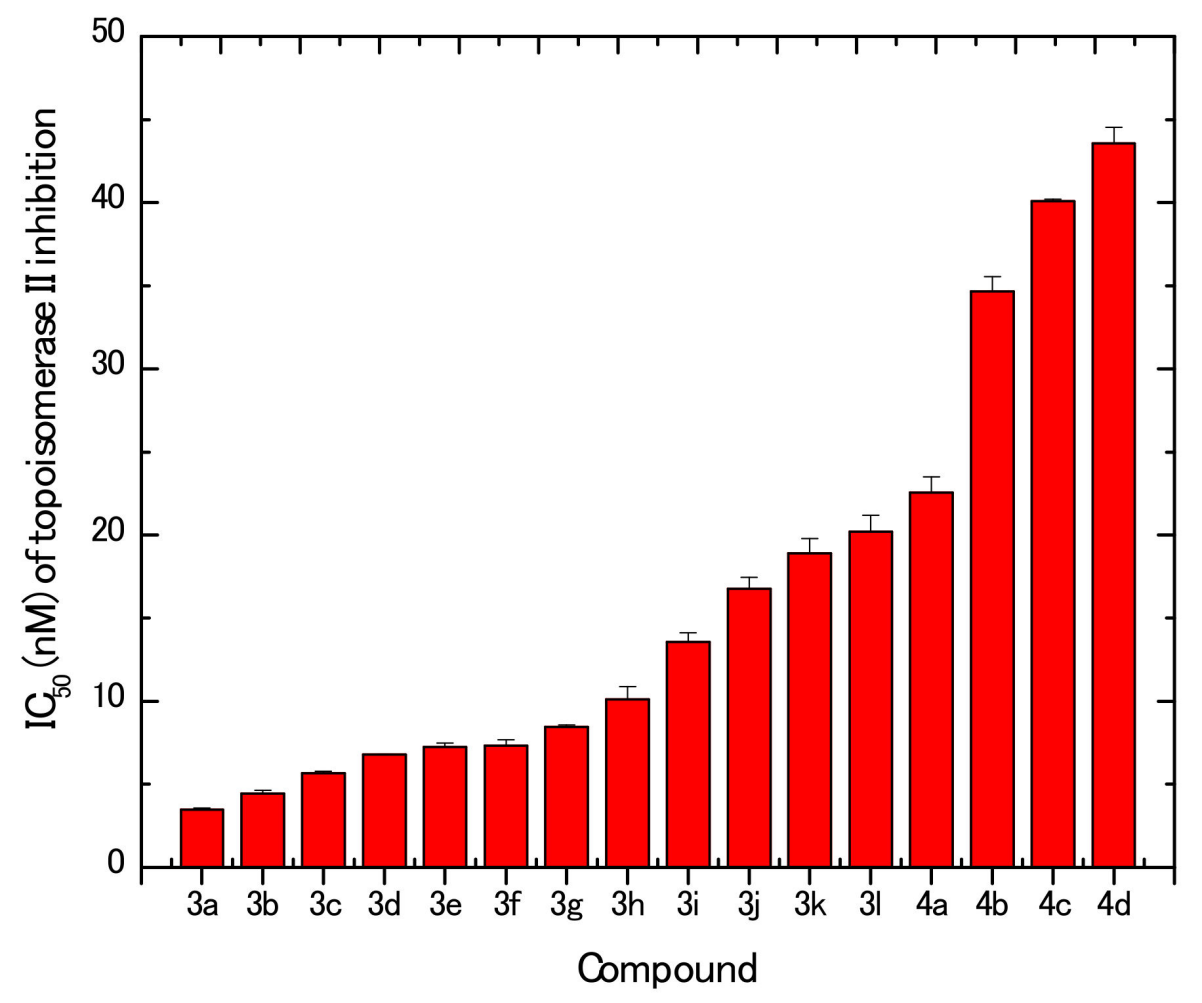
IC_50_ of Topoisomerase II Inhibitor activities of the tested compounds.

In order to further investigate the possible mechanism of action of the newly synthesized derivatives, we investigated their inhibitory effects on topoisomerase II. Results obtained (Figure 3) showed that all synthesized compounds showed potential inhibitory effects against topoisomerase II. Furthermore, the inhibitory effect was also found to follow the same descending order obtained before during in vitro and in vivo investigation: **3a**, **3b**, **3c**, **3d**, **3e**, **3f**, **3g**, **3h**, **3i**, **3j**, **3k**, **3l**, **4a**, **4b**, **4c**, and **4d**. Cpd. **3a** showed the most inhibitory action for topoisomerase II with IC_50_ value of 3.45 ± 0.13 nM, which was about 7,9% of that obtained for the lest potent compound (IC_50_ for Cpd. **4d**: 43.56 ± 0.98 nM).

#### 2.1.4. In Vitro Kinase Assay

**Figure 4:**
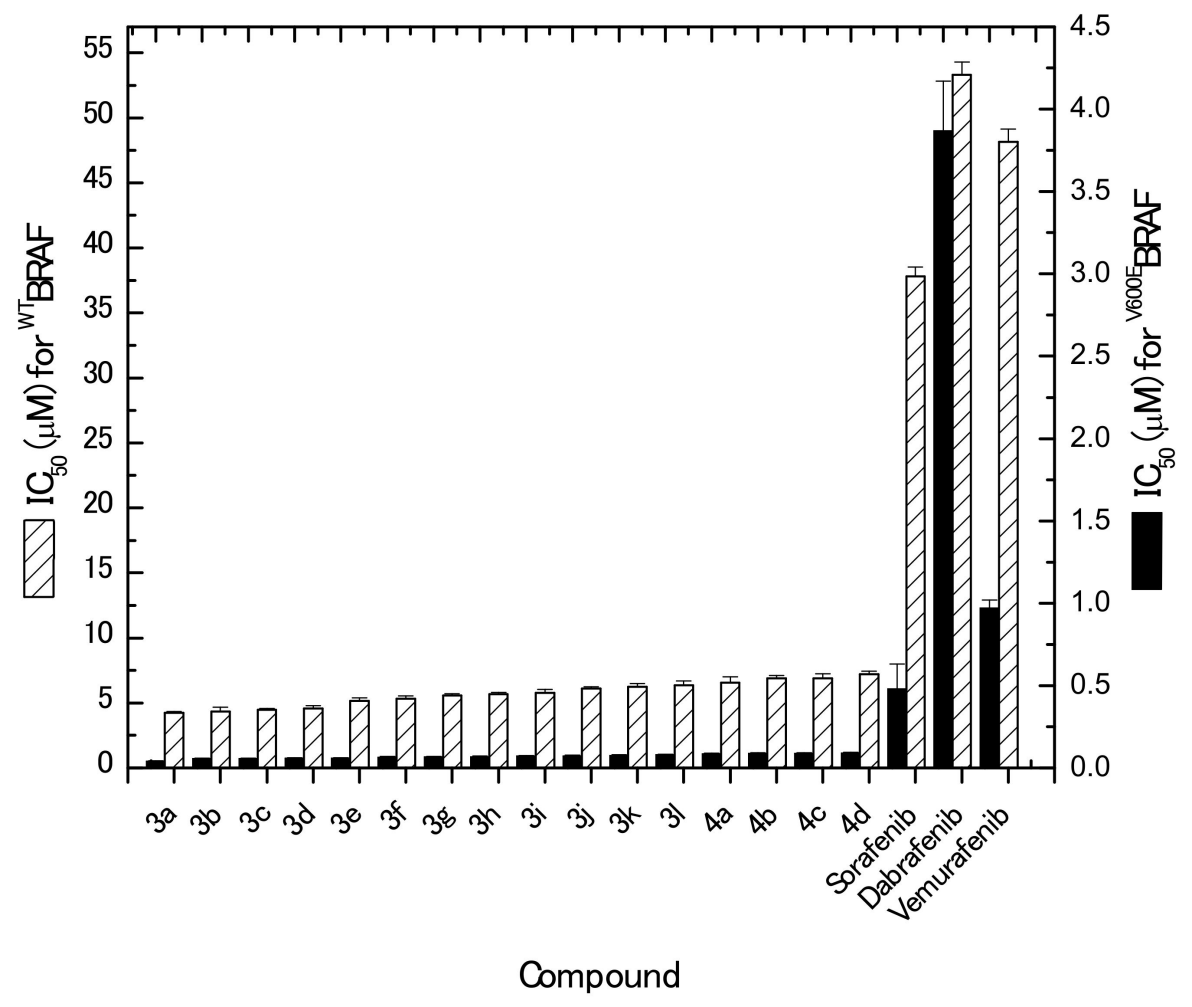
IC_50_ values of synthesized for both ^WT^BRAF and ^V600E^BRAF.

The in vitro kinase assay of synthesized derivatives was investigated against both ^WT^BRAF (BRAF kinase wild type) and ^V600E^BRAF (mutant BRAF kinase). Results obtained in Figure 4 showed that all synthesized compounds were highly active inhibitors for ^V600E^BRAF compared with moderate activities against ^WT^BRAF. Again, the descending order of activities was as follow **3a**, **3b**, **3c**, **3d**, **3e**, **3f**, **3g**, **3h**, **3i**, **3j**, **3k**, **3l**, **4a**, **4b**, **4c**, and **4d**. Compound **3a** recorded the most active inhibition (IC_50_: 0.041 ± 0.0016 and 4.23 ± 0.12 μM for mutant and wild type BRAF kinase, respectively. Furthermore, it can be seen that the inhibitory effect of Cpd. **3a** was much more superior to that obtained for different positive control drugs (0.48, 3.87 and 0.97 μM for Sorafenib, Dabrafenib and Vemurafenib, respectively.

### 2.2. Discussion

Within the framework of the current work we synthesized new estrone derivatives from their corresponding arylidines. The newly synthesized derivatives showed potential cytotoxic activities against SKOV-3 cells. Moreover, in vivo investigations revealed that the synthesized compounds were able to potentially reduce tumor volume growth over a treatment period of 45 days. 

The newly introduced 1-alkoxy benzyl moiety alone or combined with the 17- hydrazino- moities make remote destortion of the structure cage that completely elminate any hormonal activities of te estrone molceules and deviate the biological activities towards the aniticancer ones, so we investigated this property and tried to find the mechnisms of anticancer actions of these newly synthesized compounds.

In search for the possible mechanism of action of the anticancer activities of the tested compounds, we investigated the possibility of the compounds to inhibit both topoisomerase II and kinase enzymes. Results showed that the prepared compounds can potentially serve as inhibitors for these enzymes. The cytotoxic activities of the prepared estrone derivatives can be due to the inhibition of 17-hydroxysteroid dehydrogenases. We have previously reported on the anti-breast cancer activities of different estrone derivatives [23], and explained that their anticancer activities can be attributed to the inhibition of 17-hydroxysteroid dehydrogenase [24].

Concerning topoisomerase II and ^V600E^BRAF kinase inhibition, our results suggest that the newly synthesized compounds exert their cytotoxic action against SKOV-3 cancer cells by interfering with the metabolic activity of these enzymes, thus preventing cancer cells from obtaining proliferation signaling molecules essential for their growth and survival [16,17]. Different activities of the prepared compounds may be attributed to the structure activity relationship of these compounds. 

Hydrazine derivatives are generally potent than pyrazoline ones, due to their open chain structure as well as their higher electron density. Furthermore, it can be suggested that *N*-methyl derivatives are more active than *N*-phenyl ones. On the other hand, hydrazine derivatives can be divided into more active α-methoxyl derivatives and the less active α-ethoxyl, and then the least active *N*-phenyl derivatives. This can be explained due to the fact that phenyl group act as a withdrawing group. On the other hand, the weaker effect of α-ethoxyl derivatives than the α-methoxyl ones can be attributed to their bulky nature. This effect can be observed among different series of compound **3**. As the bulky nature of the substituted groups increase, accordingly more steric hindrance can be observed, leading to decreased cytotoxic activities.

## 3. Materials and Methods 

### 3.1. Chemical Synthesis

“Melting points reported are inaccurate. IR spectra were registered on Shimadzu FT-IR 8300 E (Shimadzu Corporation, Kyoto, Japan) spectrophotometer using the (KBr) disk technique. The ^1^H-NMR spectra were determined with bruker 600 MHz NMR spectrometer. The chemical shifts are expressed on the δ (ppm) scale using TMS as the standard reference. Mass spectra were recorded on Finnigan SSQ operating at 70 ev. Elemental analysis determined on a Perkin Elmer 240 (Microanalysis), Microanalysis Center, Cairo University, Cairo, Egypt”.

#### 3.1.1. Synthesis of 3-Hydroxy-16-[substituted]-estra-1(10),2,4-trien-17-ones (**2a**,**b**)

“A mixture of (**1**) (0.54 g, 20 mmol) and formaldehyde or acetaldehyde (20 mmol) in EtOH (50 mL) and aqueous KOH (10 mL, 30%) was stirred for 24 h at room temperature. The formed solid was collected by filtration, crystallized from ethanol to give compounds **2a** [25] and **2b**, respectively”.

##### *3-Hydroxy-16-[ethylene]-estra-1(10),2,4-trien-17-one* (**2b**)

Yield 97%, mp 198–200 °C, ${\left[\alpha \right]}_{D}^{25}$ = + 71 (c 1, MeOH). IR (KBr, cm^–1^): 3340 (OH), 2944 (CH, aliphatic), 1743 (C=O), 1642 (C=C). ^1^H NMR: (600 MHz, CDCl_3_): δ ppm 0.61 (1H, m, H-8β), 0.90 (3H, s, CH_3_-18), 1.01 (1H, m, H-11β), 1.12 (1H, m, H-7α), 1.15 (1H, m, H-12α), 1.25 (1H, m, H-14α), 1.44 (1H, m, H-15β), 1.63 (1H, m, H-15α), 1.72 (1H, m, H-7β), 2.01 (1H, m, H-9α), 2.13 (1H, m, H-11α), 2.50 (1H, m, H-12β), 2.56 (1H, m, H-6α), 2.66 (1H, m, H-6β), 4.90 (1H, s, OH, disappeared with D_2_O), 5.75 (1H, dd, H-2), 6.67 (1H, d, H-4), 2.08 (3H, d, CH_3_, enone), 6.41 (1H, s, enone proton), 7.12 (1H, d, H-1). ^13^C NMR: (150 MHz, CDCl_3_): δ 13.52, 21.73, 25.51, 26.64, 27.48, 29.75, 36.35, 38.61, 43.81, 48.92, 50.43, 112.79, 115.53, 126.56, 128.48, 132.50, 137.02, 138.44, 153.82, 210.02 (20 C). MS (EI): *m*/*z* 296 (100%) [M^+^]. Anal. Calcd for C_20_H_24_O_2_ (296.40): Calcd C, 81.04; H, 8.16. Found C, 81.00; H, 8.10.

#### 3.1.2. Synthesis of 16-(α-alkoxy-alkane)-17-hydrazino-estra-1(10),2,4-trien[17,16-c]-3-ol and their *N*-substituted derivatives (**3a–l**)

“A mixture of **2a,b** (14 mmol) and hydrazine derivatives, namely, hydrazine hydrate, methyl hydrazine or phenyl hydrazine (8 mmol) in absolute methanol or ethanol (30 mL) was refluxed for 5–10 h. The solvent was concentrated under reduced pressure, the formed precipitate was filtered off, washed with water, dried and crystallized from ethanol to give the corresponding derivatives **3a–l**, respectively”.

##### *16-(α-Methoxy-methane)-17-hydrazino-estra-1(10),2,4-trien-[17,16-c]-3-ol *(**3a**)

Yield 80%, mp 199–201 °C, ${\left[\alpha \right]}_{D}^{25}$ = + 81 (c 1, MeOH). IR (KBr, cm^–1^): 3444–3377 (NH, NH_2_), 3345 (OH), 2951 (CH, aliphatic), 1638 (C=C). ^1^H NMR: (600 MHz, CDCl_3_): δ ppm 0.61 (1H, m, H-8β), 0.90 (3H, s, CH_3_-18), 1.00 (1H, m, H-11β), 1.11 (1H, m, H-7α), 1.14 (1H, m, H-12α), 1.26 (1H, m, H-14 α), 1.39 (1H, m, H-15β), 1.58 (1H, m, H-15α), 1.68 (1H, m, H-7β), 2.01(1H, m, H-9α), 1.84 (1H, m, CH, 16-Hα), 2.11 (1H, m, H-11α), 2.49 (1H, m, H-12β), 2.61 (1H, m, H-6α), 2.66 (1H, m, H-6β), 2.83 (1H, d, H-17), 3.48 (3H, s, OCH_3_), 4.65 (2H, m, NH_2_, disappeared with D_2_O), 4.80 (2H, d, CH_2_-O), 4.87 (1H, s, OH, disappeared with D_2_O), 5.70 (1H, dd, H-2), 6.61 (1H, d, H-4), 7.10 (1H, d, H-1), 7.70 (1H, br.s, NH, disappeared with D_2_O). ^13^C NMR: (150 MHz, CDCl_3_): δ 13.58, 21.76, 25.57, 26.76, 28.00, 29.78, 36.38, 38.65, 43.89, 48.40, 50.45, 59.45, 69.11, 112.37, 115.56, 126.53, 132.81, 138.46, 153.84, 167.02 (20 C). MS (EI): *m*/*z* 330 (90%) [M^+^]. Anal. Calcd for C_20_H_30_N_2_O_2_ (330.46): Calcd C, 72.69; H, 9.15; N, 8.48. Found C, 72.60; H, 9.10; N, 8.40.

##### *16-(α-Methoxy-ethane)-17-hydrazino-estra-1(10),2,4-trien[17,16-c]-3-ol* (**3b**)

Yield 57%, mp. 308–310 °C, ${\left[\alpha \right]}_{D}^{25}$ = + 112 (c 1, MeOH). IR (KBr, cm^–1^): 3448-3377 (NH, NH_2_), 3348 (OH), 2959 (CH, aliphatic), 1634 (C=C). ^1^H NMR: (600 MHz, CDCl_3_): δ ppm 0.59 (1H, m, H-8β), 0.91 (3H, s, CH_3_-18), 1.01 (1H, m, H-11β), 1.12 (1H, m, H-7α), 1.14 (1H, m, H-12 α), 1.26 (1H, m, H-14 α), 1.39 (1H, m, H -15 β), 1.58 (1H, m, H-15α), 1.42 (3H, m, CH_3_,C-16 ethane), 1.69 (1H, m, H-7β), 2.02(1H, m, H-9α), 1.83 (1H, m, CH, H-16α), 2.11 (1H, m, H-11α), 2.49 (1H, m, H-12β), 2.61 (1H, m, H-6α), 2.66 (1H, m, H-6β), 2.83 (1H, d, H-17), 3.48 (3H, s, OCH_3_), 4.65 (2H, s, NH_2_, disappeared with D_2_O), 4.84 (1H, d, CH-O), 4.99 (1H, s, OH, disappeared with D_2_O), 5.72 (1H, dd, H-2), 6.61 (1H, d, H-4), 7.11 (1H, d, H-1), 7.78 (1H, bs, NH, disappeared with D_2_O). ^13^C NMR: (150 MHz, CDCl_3_): δ 13.58, 21.77, 22.22, 25.57, 26.47, 28.00, 29.78, 36.68, 38.47, 43.89, 48.69, 50.45, 59.45, 69.11, 112.37, 115.56, 126.55, 132.48, 138.46, 153.54, 167.76 (21 C). MS (EI): *m*/*z* 344 (11%) [M^+^]. Anal. Calcd for C_21_H_32_N_2_O_2_ (344.49): Calcd C, 73.22; H, 9.36; N, 8.13. Found C, 73.18; H, 9.30; N, 8.10.

##### *16-(α-Ethoxy-methane)-17-hydrazino-estra-1(10),2,4-trien-[17,16-c]-3-ol *(**3c**)

Yield 56%, mp 219–221 °C, ${\left[\alpha \right]}_{D}^{25}$ = + 77 (c 1, MeOH). IR (KBr, cm^–1^): 3445–3376 (NH, NH_2_), 3346 (OH), 2951 (CH, aliphatic), 1638 (C=C). ^1^H NMR: (600 MHz, CDCl_3_): δ ppm 0.61 (1H, m, H-8β), 0.90 (3H, s, CH_3_-18), 1.00 (1H, m, H-11β), 1.11 (1H, m, H-7α), 1.15 (1H, m, H-12α), 1.21 (3H, m, CH_3_, ethoxyl), 1.25 (1H, m, H-14α), 1.36 (1H, m, H-15β), 1.57 (1H, m, H-15α), 1.68 (1H, m, H-7β), 2.01 (1H, m, H-9α), 1.84 (1H, m, CH, H-16α), 2.10 (1H, m, H-11α), 2.50 (1H, m, H-12β), 2.60 (1H, m, H-6α), 2.70 (1H, m, H-6β), 6.61 (1H, d, H-4), 2.80 (1H, d, H-17), 3.50 (2H, m, OCH_2_), 4.70 (2H, m, NH_2_, disappeared with D_2_O), 4.80 (2H, d, CH-O), 4.87 (1H, s, OH, disappeared with D_2_O), 5.70 (1H, dd, H-2), 7.10 (1H, d, H-1), 7.70 (1H, br.s, NH, disappeared with D_2_O). ^13^C NMR: (150 MHz, CDCl_3_): δ 13.65, 18.74, 21.57, 25.17, 26.38, 28.04, 29.64, 36.54, 38.15, 44.11, 48.44, 50.54, 68.45, 69.11, 112.44, 115.67, 126.34, 131.11, 138.46, 153.84, 161.47 (21 C). MS (EI): *m*/*z* 344 (35%) [M^+^]. Anal. Calcd for C_21_H_32_N_2_O_2_ (344.49): Calcd C, 73.22; H, 9.36; N, 8.13. Found C, 73.16; H, 9.30; N, 8.10.

##### *16-(α-Ethoxy-ethane)-17-hydrazino-estra-1(10),2,4-trien-[17,16-c]-3-ol* (**3d**)

Yield 90%, mp 223–225 °C, ${\left[\alpha \right]}_{D}^{25}$ = + 95 (c 1, MeOH). IR (KBr, cm^–1^): 3440-3370 (NH, NH_2_), 3350 (OH), 2951 (CH, aliphatic), 1631 (C=C). ^1^H NMR: (600 MHz, CDCl_3_): δ ppm 0.57 (1H, m, H-8β), 0.90 (3H, s, CH_3_-18), 1.00 (1H, m, H-11β), 1.10 (1H, m, H-7α), 1.15 (1H, m, H-12α), 1.20 (3H, m, CH_3_, ethoxyl), 1.24 (1H, m, H-14α), 1.36 (1H, m, H-15β), 1.45 (3H, m, CH_3_,C-16 ethane), 1.59 (1H, m, H-15α), 1.71 (1H, m, H-7β), 1.96 (1H, m, H-9α), 1.82 (1H, m, CH, H-16α), 2.10 (1H, m, H-11α), 2.50 (1H, m, H-12β), 2.62 (1H, m, H-6α), 2.67 (1H, m, H-6β), 2.86 (1H, d, H-17), 3.48 (2H, m, OCH_2_), 4.64 (2H, s, NH_2_, disappeared with D_2_O), 4.83 (1H, d, CH-O), 4.88 (1H, s, OH, disappeared with D_2_O), 5.74 (1H, dd, H-2), 6.65 (1H, d, H-4), 7.12 (1H, d, H-1), 7.78 (1H, br.s, NH, disappeared with D_2_O). ^13^C NMR: (150 MHz, CDCl_3_): δ 13.55, 18.79, 22.07, 22.28, 25.47, 26.52, 27.77, 28.09, 36.68, 37.97, 44.09, 48.11, 51.41, 68.43, 69.13, 112.56, 115.57, 126.37, 133.18, 137.87, 155.56, 162.74 (22 C). MS (EI): *m*/*z* 358 (45%) [M^+^]. Anal. Calcd for C_22_H_34_N_2_O_2_ (358.51): Calcd C, 73.70; H, 9.56; N, 7.81. Found C, 73.64; H, 9.52; N, 7.75.

##### *16-(α-Methoxy-methane)-17-[N-methyl-hydrazino]estra-1(10),2,4-trien[17,16-c]-3-ol* (**3e**)

Yield 76%, mp 222–224 °C, ${\left[\alpha \right]}_{D}^{25}$ = + 66 (c 1, MeOH). IR (KBr, cm^–1^): 3443–3371 (NH), 3344 (OH), 2953 (CH, aliphatic), 1634 (C=C). ^1^H NMR: (600 MHz, CDCl_3_): δ ppm 0.61 (1H, m, H-8β), 0.92 (3H, s, CH_3_-18), 1.03 (1H, m, H-11β), 1.10 (1H, m, H-7α), 1.14 (1H, m, H-12α), 1.25 (1H, m, H-14α), 1.37 (1H, m, H-15β), 1.57 (1H, m, H-15α), 1.66 (1H, m, H-7β), 2.00 (1H, m, H-9α), 1.84 (1H, m, CH, H-16α), 2.13 (1H, m, H-11α), 2.32 (3H, s, NCH_3_), 2.48 (1H, m, H-12β), 2.63 (1H, m, H-6α), 2.68 (1H, m, H-6β), 2.84 (1H, d, H-17), 3.49 (3H, s, OCH_3_), 4.86 (2H, d, CH-O), 4.87 (1H, s, OH, disappeared with D_2_O), 5.72 (1H, dd, H-2), 6.63 (1H, d, H-4), 7.11 (1H, d, H-1), 7.76 (1H, brs, NH, disappeared with D_2_O), 7.99 (1H, bs, NH, disappeared with D_2_O). ^13^C NMR: (150 MHz, CDCl_3_): δ 13.53, 21.44, 25.67, 26.36, 28.03, 29.23, 36.33, 38.43, 43.83, 47.47, 48.43, 50.34, 59.35, 69.31, 112.36, 115.67, 126.52, 132.32, 138.21, 153.45, 162.32 (21 C). MS (EI): *m*/*z* 344 (45%) [M^+^]. Anal. Calcd for C_21_H_32_N_2_O_2_ (344.50): Calcd C, 73.22; H, 9.36; N, 8.13. Found C, 73.15; H, 9.31; N, 8.09.

##### *16-(α-Methoxy-ethane)-17-[N-methyl-hydrazino]-estra-1(10),2,4-trien-[17,16-c]-3-ol* (**3f**)

Yield 57%, mp 310–312 °C, ${\left[\alpha \right]}_{D}^{25}$ = + 112 (c 1, MeOH). IR (KBr, cm^–1^): 3443–3374 (NH), 3344 (OH), 2955 (CH, aliphatic), 1628 (C=C). ^1^H NMR: (600 MHz, CDCl_3_): δ ppm 0.58 (1H, m, H-8β), 0.90 (3H, s, CH_3_-18), 1.00 (1H, m, H-11β), 1.10 (1H, m, H-7α), 1.14 (1H, m, H-12α), 1.26 (1H, m, H-14 α), 1.39 (1H, m, H-15 β), 1.60 (1H, m, H-15α), 1.44 (3H, m, CH_3_, C-16 ethane), 1.70 (1H, m, H-7β), 2.00 (1H, m, H-9α), 1.82 (1H, m, CH, H-16α), 2.11 (1H, m, H-11α), 2.36 (3H, s, NCH_3_), 2.51 (1H, m, H-12β), 2.62 (1H, m, H-6α), 2.65 (1H, m, H-6β), 2.85 (1H, d, H-17), 3.44 (3H, s, OCH_3_), 4.83 (1H, d, CH-O), 4.99 (1H, s, OH, disappeared with D_2_O), 5.74 (1H, dd, H-2), 6.65 (1H, d, H-4), 7.13 (1H, d, H-1), 7.73 (1H, br.s, NH, disappeared with D_2_O), 7.99 (1H, m, NH, disappeared with D_2_O). ^13^C NMR: (150 MHz, CDCl_3_): δ 13.78, 21.54, 22.27, 25.97, 26.69, 28.03, 29.43, 36.45, 38.54, 43.68, 47.88, 48.57, 50.86, 59.46, 69.15, 112.54, 115.69, 126.46, 132.90, 138.75, 153.45, 163.60 (22 C). MS (EI): *m*/*z* 358 (100%) [M^+^]. Anal. Calcd for C_22_H_34_N_2_O_2_ (358.50): Calcd C, 73.70; H, 9.56; N, 7.81. Found C, 73.60; H, 9.49; N, 7.75.

##### *16-(α-Ethoxy-methane)-17-[N-methyl-hydrazino]-estra-1(10),2,4-trien-[17,16-c]-3-ol* (**3g**)

Yield 90%, mp 286–288 °C, ${\left[\alpha \right]}_{D}^{25}$ = + 121 (c 1, MeOH). IR (KBr, cm^–1^): 3441–3379 (NH), 3348 (OH), 2957 (CH, aliphatic), 1636 (C=C). ^1^H NMR: (600 MHz, CDCl_3_): δ ppm 0.61 (1H, m, H-8β), 0.91 (3H, s, CH_3_-18), 1.01 (1H, m, H-11β), 1.13 (1H, m, H-7α), 1.15 (1H, m, H-12α), 1.22 (3H, m, CH_3_, ethoxyl), 1.25 (1H, m, H-14α), 1.37 (1H, m, H-15β), 1.58 (1H, m, H-15α), 1.69 (1H, m, H-7β), 2.00 (1H, m, H-9 α), 1.81 (1H, m, CH, H-16α), 2.12 (1H, m, H-11α), 2.33 (3H, s, NCH_3_), 2.54 (1H, m, H-12β), 2.65 (1H, m, H-6α), 2.76 (1H, m, H-6β), 2.87 (1H, d, H-17), 3.50 (2H, m, OCH_2_), 4.85 (2H, d, CH_2_-O), 4.98 (1H, s, OH, disappeared with D_2_O), 5.74 (1H, dd, H-2), 6.65 (1H, d, H-4), 7.10 (1H, d, H-1), 7.76 (1H, br.s, NH, disappeared with D_2_O), 7.95 (1H, m, NH, disappeared with D_2_O). ^13^C NMR: (150 MHz, CDCl_3_): δ 13.35, 18.45, 21.43, 25.78, 26.80, 28.14, 29.88, 36.47, 38.90, 44.41, 47.37, 48.34, 50.54, 68.66, 69.67, 113.34, 115.55, 125.54, 131.53, 137.99, 154.44, 162.27 (22 C). MS (EI): *m*/*z* 358 (100%) [M^+^]. Anal. Calcd for C_22_H_34_N_2_O_2_ (358.50): Calcd C, 73.70; H, 9.56; N, 7.81. Found C, 73.62; H, 9.50; N, 7.74.

##### *16-(α-Ethoxy-ethane)-17-[N-methyl-hydrazino]-estra-1(10),2,4-trien-[17,16-c]-3-ol* (**3h**)

Yield 78%, mp 280–282 °C, ${\left[\alpha \right]}_{D}^{25}$ = + 135 (c 1, MeOH). IR (KBr, cm^–1^): 3441–3371 (NH), 3351 (OH), 2951 (CH, aliphatic), 1631 (C=C). ^1^H NMR: (600 MHz, CDCl_3_): δ ppm 0.60 (1H, m, H-8β), 0.93 (3H, s, CH_3_-18), 1.04 (1H, m, H-11β), 1.11 (1H, m, H-7α), 1.16 (1H, m, H-12α), 1.19 (3H, m, CH_3_,ethoxyl), 1.23 (1H, m, H-14α), 1.37 (1H, m, H-15β), 1.46 (3H, m, CH_3_,C-16 ethane), 1.61 (1H, m, H-15α), 1.72 (1H, m, H-7β), 1.82 (1H, m, CH, H-16α), 1.93 (1H, m, H-9α), 2.10 (1H, m, H-11α), 2.50 (1H, m, H-12β), 2.34 (3H, s, NCH_3_), 2.63 (1H, m, H-6α), 2.62 (1H, m, H-6β), 2.85 (1H, d, H-17), 3.43 (2H, m, OCH_2_), 4.64 (1H, s, OH, disappeared with D_2_O), 4.83 (1H, d, CH-O), 5.72 (1H, dd, H-2), 6.64 (1H, d, H-4), 7.10 (1H, d, H-1), 7.75 (1H, br.s, NH, disappeared with D_2_O), 7.88 (1H, s, NH, disappeared with D_2_O). ^13^C NMR: (150 MHz, CDCl_3_): δ 13.53, 18.67, 22.43, 22.90, 25.67, 26.78, 27.45, 28.29, 36.89, 37.65, 44.65, 47.35, 48.68, 51.90, 68.78, 69.13, 112.56, 115.56, 126.34, 133.55, 137.43, 155.34, 163.34 (23 C). MS (EI): *m*/*z* 372 (100%) [M^+^]. Anal. Calcd for C_23_H_36_N_2_O_2_ (372.54): Calcd C, 74.15; H, 9.74; N, 7.52. Found C, 74.05; H, 9.68; N, 7.48.

##### *16-(α-Methoxy-methane)-17-[N-phenyl-hydrazino]-estra-1(10),2,4-trien-[17,16-c]-3-ol* (**3i**)

Yield 67%, mp 312–314 °C, ${\left[\alpha \right]}_{D}^{25}$ = + 95 (c 1, MeOH). IR (KBr, cm^–1^): 3430–3367 (NH), 3345 (OH), 3068 (CH, aromatic), 2951 (CH, aliphatic), 1638 (C=C). ^1^H NMR: (600 MHz, CDCl_3_): δ ppm 0.61(1H, m, H-8β), 0.91 (3H, s, CH_3_-18), 1.00 (1H, m, H-11β), 1.12 (1H, m, H-7α), 1.15 (1H, m, H-12α), 1.28 (1H, m, H-14α), 1.40 (1H, m, H-15β), 1.60 (1H, m, H-15α), 1.68 (1H, m, H-7β), 2.02 (1H, m, H-9α), 1.86 (1H, m, CH, H-16α), 2.12 (1H, m, H-11α), 2.49 (1H, m, H-12 β), 2.59 (1H, m, H-6α), 2.65 (1H, m, H-6β), 2.84 (1H, d, H-17), 3.47 (3H, s, OCH_3_), 4.80 (2H, d, CH-O), 4.87 (1H, s, OH, disappeared with D_2_O), 5.70 (1H, dd, H-2), 6.61 (1H, d, H-4), 7.10 (1H, d, H-1), 7.28-7.48 (5H, m, Ar-H), 7.76 (1H, br.s, NH, disappeared with D_2_O), 7.99 (1H, s, NH, disappeared with D_2_O). ^13^C NMR: (150 MHz, CDCl_3_): δ 13.45, 21.76, 25.76, 26.67, 28.45, 29.66, 36.43, 38.65, 43.45, 48.40, 50.87, 59.45, 69.67, 112.43, 112.76, 115.66, 119.11, 126.68, 129.45, 132.67, 138.53, 152.55, 153.79, 161.02 (26 C). MS (EI): *m*/*z* 406 (77%) [M^+^]. Anal. Calcd for C_26_H_34_N_2_O_2_ (406.56): Calcd C, 76.81; H, 8.43; N, 6.89. Found C, 76.75; H, 8.40; N, 6.80.

##### *16-(α-Methoxy-ethane)-17-[N-phenyl-hydrazino]-estra-1(10),2,4-trien-[17,16-c]-3-ol* (**3j**)

Yield 66%, mp 229–231 °C, ${\left[\alpha \right]}_{D}^{25}$ = + 147 (c 1, MeOH). IR (KBr, cm^–1^): 3433–3363 (NH), 3346 (OH), 3066 (CH, aromatic), 2955 (CH, aliphatic), 1635 (C=C). ^1^H NMR: (600 MHz, CDCl_3_): δ ppm 0.62 (1H, m, H-8β), 0.92 (3H, s, CH_3_-18), 1.02 (1H, m, H-11β), 1.13 (1H, m, H-7α), 1.18 (1H, m, H-12α), 1.26 (1H, m, H-14α), 1.39 (1H, m, H-15β), 1.42 (3H, m, CH_3_, C-16 ethane), 1.58 (1H, m, H-15α), 1.69 (1H, m, H-7β), 1.83 (1H, m, CH, H-16α), 2.03 (1H, m, H-9α), 2.10 (1H, m, H-11α), 2.52 (1H, m, H-12β), 2.62 (1H, m, H-6α), 2.65 (1H, m, H-6β), 2.85 (1H, d, H-17), 3.49 (3H, s, OCH_3_), 4.86 (1H, d, CH-O), 4.89 (1H, s, OH, disappeared with D_2_O), 5.71 (1H, dd, H-2), 6.61 (1H, d, H-4), 7.12 (1H, d, H-1), 7.27-7.49 (5H, m, Ar-H), 7.77 (1H, br.s, NH, disappeared with D_2_O), 7.97 (2H, s, NH, disappeared with D_2_O). ^13^C NMR: (150 MHz, CDCl_3_): δ 13.52, 21.78, 22.92, 25.67, 26.45, 28.10, 29.90, 36.17, 38.47, 43.80, 48.69, 50.55, 59.25, 69.11, 112.12, 112.78, 115.54, 119.17, 126.46, 129.53, 132.79, 138.78, 152.42, 153.76, 163.11 (27 C). MS (EI): *m*/*z* 420 (81%) [M^+^]. Anal. Calcd for C_27_H_36_N_2_O_2_ (420.58): Calcd C, 77.10; H, 8.63; N, 6.66. Found C, 77.00; H, 8.58; N, 6.60.

##### *16-(α-Ethoxy-methane)-17-[N-phenyl-hydrazino]-estra-1(10),2,4-trien-[17,16-c]-3-ol* (**3k**)

Yield 60%, mp 338–340 °C, ${\left[\alpha \right]}_{D}^{25}$ = + 155 (c 1, MeOH). IR (KBr, cm^–1^): 3431–3362 (NH), 3343 (OH), 3064 (CH, aromatic), 2956 (CH, aliphatic), 1637 (C=C). ^1^H NMR: (600 MHz, CDCl_3_): δ ppm 0.63 (1H, m, H-8β), 0.90 (3H, s, CH_3_-18), 1.01 (1H, m, H-11β), 1.11 (1H, m, H-7α), 1.16 (1H, m, H-12α), 1.21 (3H, m, CH_3_, ethoxyl), 1.24 (1H, m, H-14α), 1.36 (1H, m, H-15β), 1.59 (1H, m, H-15α), 1.68 (1H, m, H-7β), 2.00 (1H, m, H-9α), 1.84 (1H, m, CH, H-16α), 2.11 (1H, m, H-11α), 2.50 (1H, m, H-12β), 2.61 (1H, m, H-6α), 2.70 (1H, m, H-6β), 6. disappeared (1H, d, H-4), 2.80 (1H, d, H-17), 3.53 (2H, m, OCH_2_), 4.80 (2H, d, CH-O), 4.84 (1H, s, OH, exchangeable with D_2_O), 5.70 (1H, dd, H-2), 7.15 (1H, d, H-1), 7.26-7.47 (5H, m, Ar-H), 7.76 (1H, br.s, NH, disappeared with D_2_O), 7.98 (1H, m, NH, disappeared with D_2_O). ^13^C NMR: (150 MHz, CDCl_3_): δ 13.64, 18.74, 21.57, 25.17, 26.30, 28.05, 29.64, 36.54, 38.15, 44.18, 48.46, 50.55, 68.43, 69.13, 112.49, 112.72, 115.60, 119.11, 126.34, 129.50, 131.11, 138.49, 152.40, 153.84, 162.47 (27 C). MS (EI): *m*/*z* 420 (88%) [M^+^]. Anal. Calcd for C_27_H_36_N_2_O_2_ (420.58): Calcd C, 77.10; H, 8.63; N, 6.66. Found C, 77.02; H, 8.57; N, 6.61.

##### *16-(α-Ethoxy-ethane)-17-[N-phenyl-hydrazino]-estra-1(10),2,4-trien-[17,16-c]-3-ol* (**3l**)

Yield 96%, mp 288–300 °C, ${\left[\alpha \right]}_{D}^{25}$ = + 95 (c 1, MeOH). IR (KBr, cm^–1^): 3431–3367 (NH), 3343 (OH), 3068 (CH, aromatic), 2951 (CH, aliphatic), 1631 (C=C). ^1^H NMR: (600 MHz, CDCl_3_): δ ppm 0.58 (1H, m, H-8β), 0.91 (3H, s, CH_3_-18), 1.00 (1H, m, H-11β), 1.10 (1H, m, H-7α), 1.14 (1H, m, H-12α), 1.21 (3H, m, CH_3_, ethoxyl), 1.24 (1H, m, H-14α), 1.36 (1H, m, H-15β), 1.44 (3H, m, CH_3_, C-16 ethane), 1.60 (1H, m, H-15α), 1.71 (1H, m, H-7β), 1.97 (1H, m, H-9 α), 1.83 (1H, m, CH, H-16α), 2.11 (1H, m, H-11α), 2.51 (1H, m, H-12β), 2.62 (1H, m, H-6α), 2.67 (1H, m, H-6 β), 2.88 (1H, d, H-17), 3.49 (2H, m, OCH_2_), 4.84 (1H, d, CH-O), 4.87 (1H, s, OH, disappeared with D_2_O), 5.73 (1H, dd, H-2), 6.62 (1H, d, H-4), 7.13 (1H, d, H-1), 7.23-7.47 (5H, m, Ar-H), 7.77 (1H, br.s, NH, disappeared with D_2_O), 7.87 (1H, m, NH, disappeared with D_2_O). ^13^C NMR: (150 MHz, CDCl_3_): δ 13.56, 18.77, 22.27, 22.32, 25.76, 26.78, 27.34, 28.29, 36.54, 37.90, 44.21, 48.23, 51.32, 68.34, 69.56, 112.67, 112.77, 115.56, 119.10, 126.45, 129.50, 133.47, 137.23, 152.55, 155.51, 163.73 (28 C). MS (EI): *m*/*z* 434 (93%) [M^+^]. Anal. Calcd for C_28_H_38_N_2_O_2_ (434.61): Calcd C, 77.38; H, 8.81; N, 6.45. Found C, 77.30; H, 8.75; N, 6.40.

#### 3.1.3. Synthesis of pyrazoline-3-ol derivatives (**4a–d**)

A mixture of **2a,b** (4 mmol) and hydrazine derivatives, namely, methy hydrazine or phenyl hydrazine (16 mmol) in dioxane (25 mL) was refluxed for 5 h. The mixture was evaporated under vacuum, the residue formed was triturated with water, filtered off, washed with water, dried and crystallized from MeOH to give derivatives **4a–d**, respectively.

##### *1`-Methyl-1`H-estra-1(10),2,4-trien-[17,16-c]pyrazoline-3-ol* (**4a**)

Yield 96%, mp 117–119 °C, ${\left[\alpha \right]}_{D}^{25}$ = + 109 (c 1, MeOH). IR (KBr, cm^–1^): 3351 (OH), 2946 (CH, aliphatic), 1627 (C=C), 1610 (C=N). ^1^H NMR: (600 MHz, CDCl_3_): δ ppm 0.61 (1H, m, H-8β), 0.94 (3H, s, CH_3_-18), 1.08 (1H, m, H-11β), 1.13 (1H, m, H-7α), 1.18 (1H, m, H-12α), 1.27 (1H, m, H-14α), 1.41 (1H, m, H-15β), 1.66 (1H, m, H-15α), 1.74 (1H, m, H-7β), 1.84 (1H, m, CH, H-16α), 2.01 (1H, m, H-9α), 2.10 (3H, s, NCH_3_), 2.22 (1H, m, H-11α), 2.42 (1H, m, H-12β), 2.52 (1H, m, H-6α), 2.66 (1H, m, H-6β), 2.84 (2H, d, pyrazoline-5′), 4.95 (1H, s, OH, disappeared with D_2_O), 5.75 (1H, dd, H-2), 6.65 (1H, d, H-4), 7.15 (1H, d, H-1). ^13^C NMR: (150 MHz, CDCl_3_): δ 14.60, 22.56, 25.35, 26.46, 28.77, 29.56, 36.68, 38.68, 43.34, 45.36, 48.21, 50.24, 64.45, 112.46, 115.57, 126.44, 132.56, 138.45, 152.67, 156.46 (20 C). MS (EI): *m*/*z* 310 (100%) [M^+^]. Anal. Calcd for C_20_H_26_N_2_O (310.43): Calcd C, 77.38; H, 8.44; N, 9.02. Found C, 77.29; H, 8.36; N, 8.98.

##### *1`-Methyl -1`H-5`-methyl-estra-1(10),2,4-trien-[17,16-c]pyrazoline-3-ol* (**4b**)

Yield 88%, mp 216–218 °C, ${\left[\alpha \right]}_{D}^{25}$ = + 111 (c 1, MeOH). IR (KBr, cm^–1^): 3351 (OH), 2946 (CH, aliphatic), 1627 (C=C), 1617 (C=N). ^1^H NMR: (600 MHz, CDCl_3_): δ ppm 0.62 (1H, m, H-8β), 0.92 (3H, s, CH_3_-18), 1.02 (1H, m, H-11β), 1.05 (3H, s, CH_3_-5′-methyl), 1.10 (1H, m, H-7α), 1.14 (1H, m, H-12α), 1.25 (1H, m, H-14α), 1.40 (1H, m, H-15β), 1.66 (1H, m, H -15α), 1.77 (1H, m, H-7β), 1.88 (1H, m, CH, H-16α), 2.00 (1H, m, H-9α), 2.17 (3H, s, N-CH_3_), 2.20 (1H, m, H-11α), 2.47 (1H, m, H-12β), 2.57 (1H, m, H-6α), 2.66 (1H, m, H-6β), 2.84 (1H, m, pyrazoline-5′), 4.88 (1H, s, OH, disappeared with D_2_O), 5.79 (1H, dd, H-2), 6.68 (1H, d, H-4), 7.19 (1H, d, H-1). ^13^C NMR: (150 MHz, CDCl_3_): δ 13.35, 21.80, 24.27, 25.30, 26.07, 28.20, 29.77, 36.76, 38.70, 43.67, 45.67, 48.50, 50.70, 64.45, 112.37, 115.54, 126.46, 132.40, 138.77, 153.44, 155.42 (21 C). MS (EI): *m*/*z* 324 (100%) [M^+^]. Anal. Calcd for C_21_H_28_N_2_O (324.45): Calcd C, 77.74; H, 8.70; N, 8.63. Found C, 77.66; H, 8.64; N, 8.58.

##### *1`-Phenyl-1`H-estra-1(10),2,4-trien-[17,16-c]pyrazoline-3-ol* (**4c**)

Yield 70%, mp 255–257 °C, ${\left[\alpha \right]}_{D}^{25}$ = + 128 (c 1, MeOH). IR (KBr, cm^–1^): 3347 (OH), 3071 and 3061 (CH, aromatic), 2931 (CH, aliphatic), 1633 (C=C), 1614 (C=N). ^1^H NMR: (600 MHz, CDCl_3_): δ ppm 0.60 (1H, m, H-8β), 0.90 (3H, s, CH_3_-18), 1.00 (1H, m, H-11β), 1.10 (1H, m, H-7α), 1.20 (1H, m, H-12α), 1.28 (1H, m, H-14α), 1.49 (1H, m, H-15β), 1.68 (1H, m, H-15α), 1.77 (1H, m, H-7β), 1.86 (1H, m, CH, H-16α), 2.04 (1H, m, H-9α), 2.26 (1H, m, H-11α), 2.48 (1H, m, H -12β), 2.54 (1H, m, H-6α), 2.64 (1H, m, H-6β), 2.84 (2H, d, pyrazoline-5`), 5.00 (1H, s, OH, disappeared with D_2_O), 5.71 (1H, dd, H-2), 6.70 (1H, d, H-4), 7.10 (1H, d, H-1), 7.28-7.40 (5H, m, Ar-H). ^13^C NMR: (150 MHz, CDCl_3_): δ 13.49, 21.24, 25.55, 26.46, 28.51, 29.67, 36.64, 38.45, 43.35, 50.73, 58.26, 64.45, 112.10, 112.48, 115.46, 119.31, 126.10, 129.20, 132.52, 138.79, 152.20, 153.13, 163.48 (25 C). MS (EI): *m*/*z* 372 (81%) [M^+^]. Anal. Calcd for C_25_H_28_N_2_O (372.50): Calcd C, 80.61; H, 7.58; N, 7.52. Found C, 80.52; H, 7.50; N, 7.48.

##### *1`-Phenyl-1`H-5`-methyl-estra-1(10),2,4-trien-[17,16-c]pyrazoline-3-ol* (**4d**)

Yield 75%, mp 214–216 °C, ${\left[\alpha \right]}_{D}^{25}$ = + 127 (c 1, MeOH). IR (KBr, cm^–1^): 3345 (OH), 3068 (CH, aromatic), 2949 (CH, aliphatic), 1625 (C=C), 1614 (C=N). ^1^H NMR: (600 MHz, CDCl_3_): δ ppm 0.62 (1H, m, H-8β), 0.93 (3H, s, CH_3_-18), 1.02 (1H, m, H-11β), 1.05 (3H, s, CH_3_-5`-methyl), 1.12 (1H, m, H-7α), 1.17 (1H, m, H-12α), 1.27 (1H, m, H-14α), 1.41 (1H, m, H-15β), 1.71 (1H, m, H -15α), 1.77 (1H, m, H-7β), 1.87 (1H, m, CH, H-16α), 2.02 (1H, m, H-9α), 2.22 (1H, m, H-11α), 2.46 (1H, m, H -12β), 2.54 (1H, m, H-6α), 2.65 (1H, m, H-6β), 3.83 (1H, s, pyrazoline-5`), 4.91 (1H, s, OH, disappeared with D_2_O), 5.81 (1H, dd, H-2), 6.73 (1H, d, H-4), 7.21 (1H, d, H-1), 7.28-7.48 (5H, m, Ar-H). ^13^C NMR: (150 MHz, CDCl_3_): δ 13.81, 21.89, 24.25, 25.80, 26.35, 28.34, 29.23, 36.56, 38.67, 43.34, 48.55, 50.67, 64.44, 112.70, 115.34, 119.10, 126.76, 129.50, 132.45, 138.56, 152.40, 154.36, 163.56, 164.55 (26 C). MS (EI): *m*/*z* 386 (23%) [M^+^]. Anal. Calcd for C_26_H_30_N_2_O (386.52): Calcd C, 80.79; H, 7.82; N, 7.25. Found C, 80.70; H, 7.76; N, 7.20.

### 3.2. Biological Screening

#### 3.2.1. In Vitro Cytotoxic Activities

The cytotoxic activities of the synthesized derivatives against ovarian cancer cell lines (SKOV-3) were evaluated using standard MTT assay [26,27]. SKOV-3 cells cultivated on DMEM medium supplemented with 10% FBS, 100×, 1% antibiotic/antimycotic solution and 3.6 g/L NaHCO_3_ were used. Cells were incubated under standard laboratory conditions [28,29]. Before testing compounds, cells were trypsinized, centrifuged and prepared as per our developed protocol [30,31]. MTT assay relies on the enzymatic conversion of the MTT substrate into purple formazan by living cells mitochondrial enzymes. Shortly, 96 well plates were seeded (10,000 cells/100 µL/well), then incubated for 24 h at standard conditions. Afterwards, cells were exposed to different concentrations from prepared compounds and incubated for another 24 h. Thereafter, MTT (10 µL, 5 mg/mL, PBS) were added to each well, and plates were incubated for 4 h. Supernatants were then replaced discarded with 200 µL of DMSO to dissolve the precipitated formazan crystals. Formazan absorbance, proportional to living cell number, was read at 550 nm using a microplate reader. Viability percentages were calculated and the corresponding IC_50_ values were obtained from the linear regression of the calibration curve. For comparison, doxorubicin (DOX) and resveratrol (RES) were used as positive controls. Furthermore, the effect of the newly synthesized derivatives was tested against normal fibroblast cell line to assess their toxicities. 

#### 3.2.2. In Vitro Anti-Ovarian Xenograft Model

Anti-ovarian cancer xenograft animal model was developed in nude mouse using SKOV-3 cells according to McCauley et al. [32]. Treatment protocol was approved by the University of South Dakota, Institutional Animal Care and Use Committee (study protocol 50-01-05-08B). Immunodeficient (athymic nude-Foxn1nu) female mice were inoculated (1 × 10^6^ cells/site). Upon tumor development, (av. volume ~50 mm^3^), 10-mice groups were implanted s.c. with constant release pellets containing tested compound (1 µM/gm) or placebo. Animal and tumor development were monitored measured every 5 days after pellet implantation, using standardized Caliper in two perpendicular diameters of the implant according to our previous work [2]. Due to large tumor volumes, (5-fold difference between largest and smallest tumor), relative tumor volumes were used for comparison. Relative tumor volume = Vt_volume at time t_/V0_initial volume_. 

#### 3.2.3. Topoisomerase II Inhibition

The inhibitory effects of the synthesized compounds against topoisomerase II were investigated using relaxation assay according to Goyeneche, et al. [33]. Topoisomerase II was purified from P388 cells, where enzyme unit corresponds to the activity required complete relaxation of 0.125 g of supercoiled pBR-322 DNA at 30 °C for 1 h. The assay was performed as per protocol and samples were subjected to electrophoresis (0.7% agarose gels, TBE buffer. Ethidium bromide-stained DNA was examined under UV light.

#### 3.2.4. In Vitro Kinase Inhibition 

##### Protein Expression and Purification

BRAF wild-type and V600E mutant kinase domains were expressed and purified according to Nakamura et al. [34]. Shortly, Sf 9 cells infected with BRAF kinase domain containing baculovirus were resuspended, sonicated, and then cleared using ultracentrifugation. Afterwards, the cleared lysate with mixed with equilibrated Talon resin, and the resin then washed with 10 column volumes of wash buffer and then eluted with buffer (25 mM Tris, pH 7.0, 250 mM NaCl, 160 mM imidazole, 10% glycerol). The eluant was then diluted, concentrated using Superdex 200 gel filtration column, purified as previously described and finally stored at –80 °C until use.

##### In Vitro ELISA-Based Kinase Assay

“This method was adopted from Nakamura et al. [34] and Qin et al. [35]. The previously prepared GST MEK-His protein was diluted in TTBS buffer (100 μL, 50 μg/mL), and then bound to 96-plate wells coated with glutathione (Pierce Biotechnology). Two DMSO-dilutions of each compound (1 µL) were added to 50 μL (50mM HEPES, pH7.0, 0.7 pmol of BRAF^V600E^ kinase). Tubes were incubated at ambient temperature for 1 h, then added to GST-MEK-His-plate wells. Afterwards, 50 μL of phosphorylation buffer were added to start kinase reaction (37 °C/30 min/shaking). Reaction was stopped, then substrate was added, and the signal was recorded. The high throughput inhibitor screening was carried out according to standard assay conditions at different concentrations, to generate a sigmoidal dose response curve, a four-parameter logistic model using GraphPad Prism, for BRAF proteins, which was used to obtain corresponding IC_50_ values”.

#### 3.2.5. Statistical Analysis

“Measurements were carried out in thrice and data are represented as means ± SEM. Significance was calculated by Student’s t-test using SPSS software (SPSS Inc., Paris, France). ^*^ *p* <0.05, ^**^ *p* < 0.01, ^***^ *p* < 0.001. IC_50_ values were calculated using GraphPad Prism software, GraphPad Software, San Diego, CA, USA [36]”.

## 4. Conclusions

During the current investigation, we synthesized a series of 17-hydrazino- and 17,16-c-pyrazoline estrone derivatives. Prepared compounds exhibited potential in vitro and in vivo anti-ovarian cancer activity against SKOV-3 cells. Compound **3a** was found to be the most potent with an IC_50_ value of 4.23 ± 0.12 nM. Additionally, in vivo xenograft ovarian cancer model showed that Cpd. **3a** was able to reduce tumor growth by 93.61 ± 0.7% after 40 days of treatment. Furthermore, the newly synthesized compounds were able to interfere with tumor proliferation through inhibiting the activity of topoisomerase II and ^V600E^BRAF, where the obtained IC_50_ values for both enzymes were 3.45 ± 0.13 nM and 0.041 ± 0.0016 μM, respectively.
